# International partnerships in infectious diseases research, training, and control.

**DOI:** 10.3201/eid0707.017721

**Published:** 2001

**Authors:** J Breman, J LeDuc

**Affiliations:** National Institutes of Health, Bethesda, Maryland 20892-2220, USA. jbreman@nih.gov


### International Partnerships in Infectious Diseases Research, Training, and Control

**Joel Breman* and James LeDuc†**
*National Institutes of Health, Bethesda, Maryland, USA; †Centers for Disease Control and Prevention, Atlanta, Georgia, USA

Partnerships are sine qua nons for effective work in international health. While individuals, institutes, and agencies comprise the usual coalitions, linkages between research, training, and control activities are also essential in international science and public health, and a balance between these components must be fostered. Support for research is particularly important when effective disease control interventions do not exist or are not available for managing emerging or reemerging infectious diseases. The five presentations in this panel represent outstanding examples of the need for close links between research, training, and control activities.

In 1998, the Burroughs Wellcome Fund (BWF) and the Wellcome Trust launched a joint research effort focused on infectious diseases of the tropical developing world. This grant program addresses parasitic, bacterial, fungal, and non-HIV/AIDS related viral infections of importance to developing tropical countries and their collaborators in developed nations. The North American and United Kingdom institutions that have funded projects must make the tropics their center of operations. The program is an experiment for the fund, allowing the BWF to explore a new, collaborative approach to health philanthropy compared to prior experience when focus was on a specific scientific topic, often investigated outside of tropical areas. (See Victoria McGovern's article in this issue on p. 564.)

The Fogarty International Center (FIC) of the National Institutes of Health (NIH) advances health research through international scientific cooperation and is the center for NIH international activities. The Multilateral Initiative on Malaria (MIM) is an alliance of organizations and individuals that aim to facilitate international collaboration and cooperation in scientific research that will lead to the control of malaria. The rotating secretariat of MIM was moved from the Wellcome Trust to the FIC in 1999 on recommendation of the partners. To ensure that research findings are applied to malaria treatment and control, scientists in malaria-endemic countries must be at the forefront of research addressing the local malaria situation.

MIM supports research that will lead to better use of current control methods and development of new and sustainable methods of malaria control in endemic countries. MIM works to strengthen and sustain malaria research capacity in endemic countries through regional and international scientific collaboration and training. It promotes regional and international communication and cooperation to maximize the impact of resources and to avoid the duplication of effort. MIM also aims to facilitate dialogue between researchers and control program personal in malaria-endemic countries to promote research that will address the needs of malaria control programs and eventually encourage collaborative research between these two groups. Finally, MIM facilitates communication among scientists, public health professionals, and policymakers to ensure that research findings lead to policy changes at the government and international levels.

The research grant component of MIM remains with the Special Programme for Research and Training in Tropical Diseases (TDR)/World Health Organization program. The task force on malaria Research Capability Strengthening (RCS) in Africa, coordinated by the United Nations Development Programme/World Bank and WHO Special Programme for Research and Training in Tropical Diseases (TDR), represents a collaborative funding strategy involving multiple agencies and governments to promote capacity-building activities carried out by MIM in Africa. (See Fabio Zicker's article in this issue on p. 529.)

Members of the East African AIDS Training Initiative have developed a model for HIV/AIDS education and training for community-based health- care workers at the grass roots level. The goal was to implement a community-owned program which could be readily adapted for the needs of any resource-poor community. Two factors led to developing the program. First, requests were received for education and training from members of the health-care community in Nairobi; second, education and training delivered at the grass roots level is believed to be the most effective vehicle for introducing rapid social change. This program involved a 3-day residential workshop and continues to be monitored with quarterly site visits in support of participants. Outcomes demonstrate the positive effects of partnerships among community members, funding organizations, and individual charitable donors. The careful development of individual action plans coupled with ongoing support of training mentors via site visits has contributed to the success of this program.

The International Trachoma Initiative is focusing on the world's leading cause of preventable blindness. An estimated 6 million people are blind or visually impaired due to trachoma, and an additional 150 million have the disease. Trachoma is an infectious disease caused by the bacterium *Chlamydia trachomatis*. The disease is most common in children but causes blindness in adults, particularly women. Poverty is the fundamental determinant of trachoma. It results in a lack of basic sanitation, medical care, drugs, and education on prevention and cure in trachoma-endemic areas. Pfizer, Inc. and the Edna McConnell Clark Foundation founded the International Trachoma Initiative (ITI) in

Address for correspondence: Joel Breman, Fogarty International Center, NIH, Bldg. 31, Room B2C39, 31 Center Drive, MSC 2220, Bethesda, Maryland 20892-2220, USA; fax: 301-402-0779, e-mail: jbreman@nih.gov

November 1998 with the explicit mission of working to advance the elimination of trachoma and the blindness it causes. A WHO-approved strategy called SAFE is simple, sustainable, and addresses both cure and prevention:

Surgery for trichiasis—the immediate precursor to blindness
Antibiotics to treat active disease
Facial cleanliness to reduce transmission
Environmental improvement to control the agents of the disease

In ITI countries the antibiotic used is Zithromax (azithromycin), donated by Pfizer. A single oral dose of Zithromax once a year is as effective as the standard treatment of tetracycline eye ointment 2 times a day for 6 weeks. The ITI is currently working in five countries: Morocco, Tanzania, Mali, Ghana, and Vietnam. The ITI works with ministries of health to devise an operating plan and joins WHO, United Nations Children's Fund, and nongovernmental organizations to carry out this work.

**Acknowledgments**

We thank the following panelists for their outstanding contributions: Victoria McGovern, Gerald Keusch, Fabio Zicker, Natasha Martin, and Joseph Cook.

### Emerging Infectious Diseases and the Law

**Edward P. Richards**
University of Missouri-Kansas City, Kansas City, Missouri, USA

In the 1960s, the United States began to lose interest in public health. The development of effective vaccines and antibiotics, combined with the long-term benefits of sanitary reforms begun 100 years earlier, fostered the belief that communicable diseases had been conquered and that it was time to focus the nation's resources on chronic diseases such as cancer and heart disease. This shift led to the deterioration of the public health infrastructure, including public health law training and practice. At the same time, bioethics and the legal specialty of health law began to evolve. Both of these fields were individual-centered: bioethics concentrated in individual autonomy and health law concentrated on the delivery of, and reimbursement for, personal health services. By the 1980s, legal discourse and training on health and public health was dominated by an individual-centered jurisprudence that subordinated the public's interest to that of the individual. Although this approach resulted in important advances in patient autonomy, it undermined the public's understanding and acceptance of the traditional role of public health law—the protection of the health of the population. Many states weakened their communicable disease-reporting laws and otherwise made it more difficult to identify and manage communicable disease threats. More critically, public health professionals began to believe that they do not have the legal authority to restrict individual behavior to protect the public health and that their role is to provide personal health services on the same basis as private health care providers.

The threat of emerging infectious diseases and bioterrorism is forcing the states and the federal government to reassess the U.S. public health infrastructure and the provision of public health services, as well as to review international treaties and trade agreements to ensure that they are consistent with effective public health measures. As part of this process, it is critical to ensure that each jurisdiction has adequate legal authority to protect the health of the public and to act quickly in the face of bioterrorism or a disease outbreak. This will require the restoration of more traditional public health laws in some jurisdictions and the training of lawyers, judges, and public health professionals in public health jurisprudence. The federal government should help coordinate state efforts and should ensure that there are no federal law impediments to effective public health enforcement.

The restoration and expansion of the public health infrastructure and the development of more effective public health legal services will have many benefits beyond improving the response to emerging infectious diseases and bioterrorism. Achieving these goals is also essential to the improvement of the delivery of routine public health services such as food sanitation, immunizations, and the abatement of hazardous environmental conditions.

Dr. Richards is executive director of the Center for Public Health law at the University of Missouri-Kansas City School of Law. His research interests focus on public health and biotechnology. For more information on public health law and practice, contact the Center for Public Health Law through its Web site: http://plague.law.umkc.edu/cphl.

Address for correspondence: Edward P. Richards, Center for Public Health Law, University of Missouri-Kansas City School of Law, 5100 Rockhill Road, Kansas City, MO 64110, USA; fax: 816-235-5276; e-mail: richardse@umkc.edu

